# Quality in general practice – state of affairs or dynamic process?

**DOI:** 10.1080/02813432.2024.2379481

**Published:** 2024-08-16

**Authors:** Torunn Bjerve Eide, Torsten Risør

**Affiliations:** a Department of General Practice, Institute of Health and Society, University of Oslo, Oslo, Norway; b Department of Community Medicine, UiT The Arctic University of Norway, Tromsø, Norway

*Quality*. A seemingly simple word, but with a multitude of interpretations and connotations. The concepts of quality and quality improvement (QI) are increasingly important in discussions regarding general practice and primary care. As general practitioners (GPs), we have an unspoken ambition to do our work as well as possible for as many patients as possible, believing this to reflect high quality practice. In many countries, however, pressure increases to use quality measures as ways to monitor and direct our medical services. Such measures also become subject for political debate about prioritization and efficiency. The simpleness of the word *quality* is long gone and the present landscape contains both tensions and uncertainties as yet unresolved.

The Nordic Colleges of General Practice list QI among the core values of general practice [1]. To obtain good quality in any part of the health services, the available framework in terms of financial and human resources must allow for QI. Furthermore, culture and motivation in healthcare strongly affect the possibility of achieving good QI work [2,3]. In a 2022 study, Norwegian GPs reported that the desire to be a better doctor and provide evidence-based services were important motivating factors for participating in a QI program [4]. Time was reported a restricting factor, but GPs were very positive toward discussing clinical data from their own practices with other colleagues. All of this indicates that GPs, as a group, have a pervasive goal to provide good quality health services.

However, the perspective in general practice and from health authorities may not align completely. In Denmark, the regional clinical quality databases (RKKP) provide extensive information on hospital productivity and quality. Such systematic disease specific data can be useful as a QI tool for specialized hospital departments, but may not translate to good quality indicators for general practice. The quality committee of the Danish College of General Practice (DSAM) often has to argue how the reality of general practice can make simple quality measurements difficult.^1^ Multimorbid patients who may not follow GPs’ advice, who are treated at many different levels of the health services and who have extensive medication lists, pose a challenge for development of consistent quality measuring tools that capture the most important features of general practice. Such tools must also take into account available local services (or lack thereof), as well as patients’ resources and motivations to engage in e.g. lifestyle changes. As much as we all wish for high quality services, it is apparent that our understanding of what this is and how it may be measured differs.

In British healthcare services, the system of Quality and Outcomes Framework (QOF) has since 2004 served as a way to financially incentivize high quality services. Through QOF, data are harvested at the level of each GP practice, and the data are accessible for the public. It has improved easy access to clinical data, allowing for activity monitoring as well as research. However, the framework does not capture or reward, e.g. the extent of person-centered care or continuity of care, both essential features of general practice services [5].

In Norway, the Directorate of Health generates quality indicators for different parts of the health care system, including primary care services [6]. They are not used as a basis for sanctions but are available as a tool for identifying areas in need of change or improvement. The indicators are calculated per county and nationally, but not on the level of individual GPs or practices. They can be used by either municipalities or GPs to compare their own situation to others, for instance regarding prescription rates to the elderly, or the patients’ experiences with GP contacts. However, they do not provide a system for extracting individual data, or for using the information in structured QI programs.

On the level of the individual clinic, quality is about how to run and develop the clinic as both an effective clinical arena and as a workplace. In Denmark, the recent invention of clusters – ‘klynger’, similar to the international term quality circles – has quickly become a success. The initiative provides GPs in a cluster with access to structured data from their own clinic, compared with neighboring practices’ data. Rather than participants trying to adjust one’s data toward the mean for the cluster, it has instead become a welcome opportunity for GPs to reflect critically on their own data and their own practices. Becoming aware of what they do in everyday practice becomes more important than adjusting toward the mean [7].

In Norway, a similar approach is applied in several of the programs that are provided by the Norwegian Centre for Quality Improvement in Medical Practices (SKIL), which encourages the use of GPs’ CME groups as arena for QI work. The well received QI program RAK (Correct antibiotic use in the primary care services) gives GPs access to their own practice data within a structured, group-based QI program, providing the opportunity to see change over time following course participation.

If you add control to quality, the focus changes from learning and reflection toward making sure that certain standards are met, possibly with consequences for the people involved when they are not. When *development* of quality is the goal, the engagement of people is key and is usually about all the people working in a clinic; not just about how they perform as individuals, but how they perform as a team. There is therefore a potential tension between quality development and quality control. Quality initiatives, whether they stem from professional organizations or from policymakers, must balance development and control and take into consideration the particularities of general practice.

*Quality* as a term is thus misleading in its apparent simpleness. In the book ‘Zen and the art of motorcycle maintenance’ by Robert Pirsig [8], the protagonist spends much of the book wondering about what quality entails, a key question being if it is a noun – something that can be defined – or a verb – something that we engage in. Without spoiling the drama of the book, we can reveal that he does arrive at an answer to this question. We believe that we, as GPs, need to discuss the same question and to reach our own answer.

## Note

1 Coauthor Torsten Risør is a member of the DSAM quality committee since 2022.
